# Coincident Activity of Converging Pathways Enables Simultaneous Long-Term Potentiation and Long-Term Depression in Hippocampal CA1 Network *In Vivo*

**DOI:** 10.1371/journal.pone.0002848

**Published:** 2008-08-06

**Authors:** ZhiFang Dong, HuiLi Han, Jun Cao, Xia Zhang, Lin Xu

**Affiliations:** 1 Key Laboratory of Animal Models and Human Disease Mechanisms, Kunming Institute of Zoology, Chinese Academy of Sciences, Kunming, People's Republic of China; 2 University of Ottawa Institute of Mental Health Research, Ottawa, Ontario, Canada; 3 Mental Health Institute, Second Xiangya Hospital of Central South University, Changsha, People's Republic of China; National Institutes of Health, United States of America

## Abstract

Memory is believed to depend on activity-dependent changes in the strength of synapses, e.g. long-term potentiation (LTP) and long-term depression (LTD), which can be determined by the sequence of coincident pre- and postsynaptic activity, respectively. It remains unclear, however, whether and how coincident activity of converging efferent pathways can enable LTP and LTD in the pathways simultaneously. Here, we report that, in pentobarbital-anesthetized rats, stimulation (600 pulses, 5 Hz) to Schaffer preceding to commissural pathway within a 40-ms timing window induced similar magnitudes of LTP in both pathways onto synapses of CA1 neurons, with varied LTP magnitudes after reversal of the stimulation sequence. In contrast, in urethane-anesthetized or freely-moving rats, the stimulation to Schaffer preceding to commissural pathway induced Schaffer LTP and commissural LTD simultaneously within a 40-ms timing window, without affecting synaptic efficacy in the reversed stimulation sequence. Coincident activity of Schaffer pathways confirmed the above findings under pentobarbital and urethane anesthesia. Thus, coincident activity of converging afferent pathways tends to switch the pathways to be LTP only or LTP/LTD depending on the activity states of the hippocampus. This network rule strengthens the view that activity-dependent synaptic plasticity may well contribute to memory process of the hippocampal network with flexibility or stability from one state to another.

## Introduction

The hippocampal network plays critical roles in certain types of learning and memory[1], [2]. It is widely believed that the cellular mechanism underlying memory is activity-dependent synaptic plasticity[3]–[5], e.g. *N*-methyl-D-aspartate receptor (NMDAR)-dependent forms of long-term potentiation (LTP) and long-term-depression (LTD). Most of the experimental studies used high-frequency stimulation (HFS) to induce LTP[6] and low-frequency stimulation (LFS) to evoke LTD[7], [8]. This view is further broadened by Levy and Steward's finding in which either LTP or LTD in the contralateral pathway to dentate gyrus is determined by the sequence of coincident ipsilateral/contralateral perforant activity at 400 Hz within a 20-ms window[9]. It provides a timing basis for synaptic plasticity[10], which is termed as spike timing-dependent plasticity (STDP) because the sequence of coincident pre- and postsynaptic spiking within a few tens of milliseconds determines either LTP or LTD in the pathway, respectively[11], [12].

These powerful mechanisms of memory may also cause stability problems in a neural network[13]. A balance of LTP and LTD is essential for the stability of a neural network as those demonstrated in the amygdala *in vitro* [14]. However, this balance is difficult to achieve if LTP and LTD are induced independently rather than simultaneously[13]. Also, another stability problem may occur if LTP or LTD of the synapses increases the likelihood that they will be further strengthened or weakened[13]. Furthermore, a neural network will meet a dilemma in synaptic modification if the stability is required for retaining acquired information as well as the flexibility for learning new information[15]. However, according to Wiener's control theory, the stability problems can be avoided if the stability or flexibility of a neural network is dynamically switched by the state of itself. Since the hippocampal network is critical for certain types of memory[1], [2], it is very important to address whether and how LTP and LTD are induced simultaneously *in vivo*, a condition essential to address whether and how the state of the network itself affects the induction.

**Figure 1:**
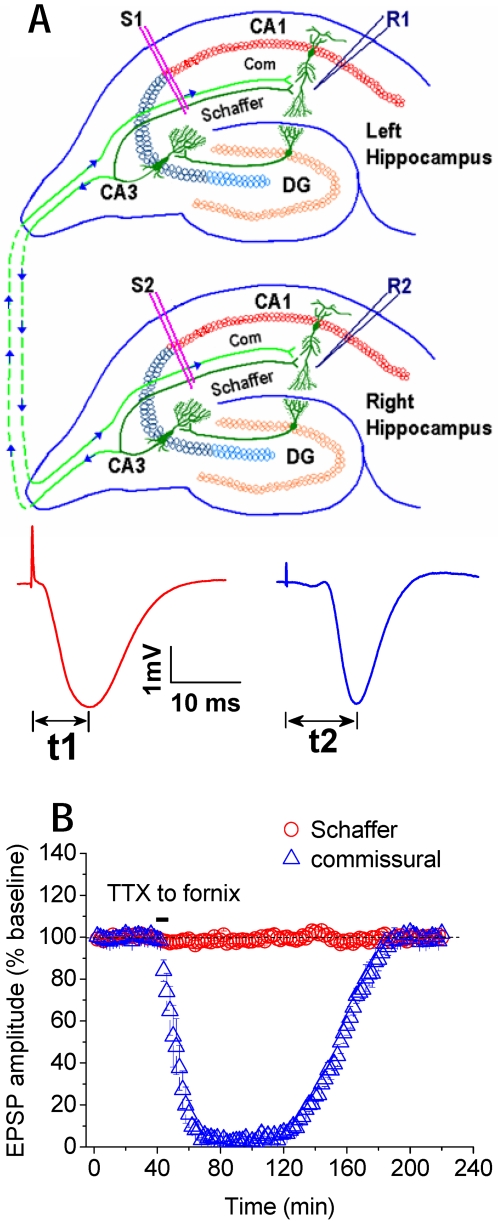
Schaffer and commissural pathways in CA3-CA1 network. A, Recording electrode (R1) in CA1 stratum radiatum records field excitatory postsynaptic potentials (fEPSPs) in response to stimulation of ipsilateral Schaffer (S1) and contralateral commissural pathway (S2) at stratum radiatum, respectively. Schaffer = Schaffer collaterals; Com = commissural fibers. B, Peak latency of Schaffer fEPSP (t1) is about 5 ms shorter than that of commissural fEPSP (t2). Timing window (Δt) between Schaffer and commissural inputs is calculated from the inter-peak intervals (t2−t1). Infusing tetrodotoxin (TTX), the sodium channel blocker, bilaterally into the fornix where commissural fibers cross the midline suppressed the fEPSP from commissural pathway, but had no effect on the fEPSP from Schaffer pathway (n = 4).

The hippocampal network of ipsilateral Schaffer/contralateral commissural inputs determines an intrinsic timing because the peak latency of the field excitatory postsynaptic potentials (fEPSP) is longer in the commissural than in the Schaffer input within ∼5 ms (Fig. 1A). Thus the timing of the afferent pathways can be calculated by the inter-peak intervals of the fEPSPs (Δt = t2−t1, Fig. 1B). Furthermore, it is known that each excitatory input can trigger postsynaptic neurons to fire spikes for a few milliseconds (4–6 ms) with ∼ 8–15 ms peak latency[16], [17]. Accordingly, we proposed a stability hypothesis that coincident Schaffer and commissural activity could cause LTP and LTD simultaneously in both pathways converging onto CA1 synapses of the hippocampal network, the state of which could also affect the induction, thereby contributing to memory process of the network.

**Figure 2:**
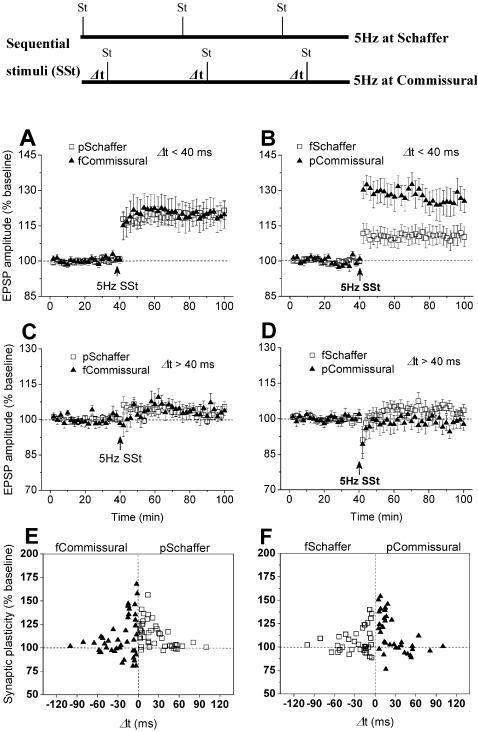
Coincident Schaffer/commissural activity induces LTP in both pathways in pentobarbital-anesthetized rats. A, Sequential stimulation (600 pulses, 5 Hz for 2 min) to Schaffer preceding to commissural pathway within a timing window of 40 ms induced LTP in both pathways (n = 23; pSchaffer: 119.3±5.8%; fCommissural: 120.3±3.2%; P<0.001 vs. baseline of pSchaffer or fCommissural; P = 0.476 pSchaffer vs. fCommissural). p = preceding; f = following. B, When the activity sequence was reversed and within the timing window of 40 ms, the stimulation induced larger LTP in the commissural and smaller LTP in the Schaffer pathway (n = 19; fSchaffer: 110.9±5.2%; pCommissural: 126.4±4.1%; P<0.001 vs. baseline of fSchaffer or pCommissural; P = 0.015 fSchaffer vs. pCommissural). C, The stimulation to Schaffer preceding to commissural pathway outside the 40-ms window (Δt = 40 to 100 ms) failed to affect synaptic efficacy in either pathway (n = 11; pSchaffer: 105.4±1.8%, P = 0.065 vs. baseline; fCommissural: 103.7±2.2%; P = 0.085 vs. baseline; P = 0.338 pSchaffer vs. fCommissural). D, Similarly, the stimulation to Schaffer following to commissural pathway beyond the 40-ms window (Δt = 40 to 100 ms) had no significant effect on synaptic efficacy in either pathway (n = 10; fSchaffer: 102.4±2.9%, P = 0.055 vs. baseline; pCommissural: 98.6±2.5%, P = 0.091 vs. baseline; P = 0.068 fSchaffer vs. pCommissural). E and F, Individual data confirmed the findings that the stimulation to Schaffer and commissural pathway by one preceding the other within about 40-ms timing window effectively evoked LTP in CA1 in the both pathways.

A prominent state of the hippocampal network is theta activity at 4–8 Hz in rat *in vivo* [18], which is associated with synaptic modifications of CA3-CA1 synapses in freely-moving rats[19]. Thus, to test the stability hypothesis, we tried a novel strategy to induce LTP and LTD by giving sequential stimulation (SSt, 600 pulses at 5 Hz) to Schaffer preceding or following to commissural pathway within a few tens of milliseconds (see *insets*, Fig. 2), in pentobarbital- and urethane-anesthetized rats. Furthermore, in freely-moving rats, we used a tetra-pathway technique to record Schaffer and commissural fEPSP from both the left and right CA1s, because in the same rat, when SSt was applied to Schaffer preceding commissural pathway (e.g. Δt = 40 ms) and the fEPSP was recorded on the left CA1, the activity sequence caused by the same SSt was naturally reversed on the right CA1, i.e. Schaffer following commissural pathway (Δt≈35 ms, due to ∼5 ms difference in peak latency). The prominent theta state of the hippocampal network under these experimental conditions was monitored by EEG from the hippocampal recording electrode. Finally, we further confirmed the above findings using SSt to independent Schaffer pathways by one preceding the other under pentobarbital and urethane anaesthesia.

## Results

### Coincident Schaffer/commissural activity induced LTP in both pathways in pentobarbital-anaesthetized rats

Using pentobarbital anaesthesia and techniques described previously[20]–[22], we first identified independent Schaffer and commissural fEPSPs that converge on the same recording electrode in the CA1 stratum radiatum as indicated by the lack of paired-pulse interaction[19]–[21], [23]. To further confirm the independence of Schaffer and commissural fEPSPs, we infused the sodium channel blocker tetrodotoxin (TTX) bilaterally into the fornix, where hippocampal commissural fibres cross the midline. TTX rapidly blocked the commissural fEPSPs for few hours without affecting the Schaffer fEPSPs (see Fig. 1B).

After a stable baseline for at least 40 min was obtained, sequential stimulation (SSt, 600 pulses at 5 Hz) applied to Schaffer preceding to commissural pathway within a 40-ms timing window (Δt<40 ms) induced similar magnitudes of LTP in both pathways (Fig. 2A). When the activity sequence was reversed (i.e. SSt to Schaffer following to commissural activity), the SSt induced a larger LTP in commissural and a smaller LTP in Schaffer pathway (Fig. 2B). To our knowledge, this is the first example for LTP simultaneously induced in both these pathways by coincident Schaffer and commissural activity at 5 Hz. Further studies showed that this type of activity-dependent LTP required a narrow timing window, similar to those demonstrated in STDP studies[11], [12]. First, SSt when Schaffer preceded or followed commissural pathway beyond that timing window (Δt = 40 to 100 ms) had no significant effect on synaptic efficacy in either pathway (Fig. 2C, D). Similar results were also indicated by a distribution plot for individual experiments at either Schaffer preceding or following commissural input (Fig. 2E, F). Second, SSt to either Schaffer or commissural pathway alone (i.e. Δt = 60 min) failed to affect synaptic efficacy in either pathway (see Fig. S1A). Third, SSt at 5 Hz is also critical for the induction of synaptic plasticity because SSt at either 1 or 10 Hz to Schaffer preceding to commissural pathway (Δt<40 ms) had no effect on synaptic efficacy in either pathway (see Fig. S1B, C).

### Coincident Schaffer/commissural activity enabled Schaffer LTP and commissural LTD in urethane-anaesthetized or freely-moving rats

**Figure 3:**
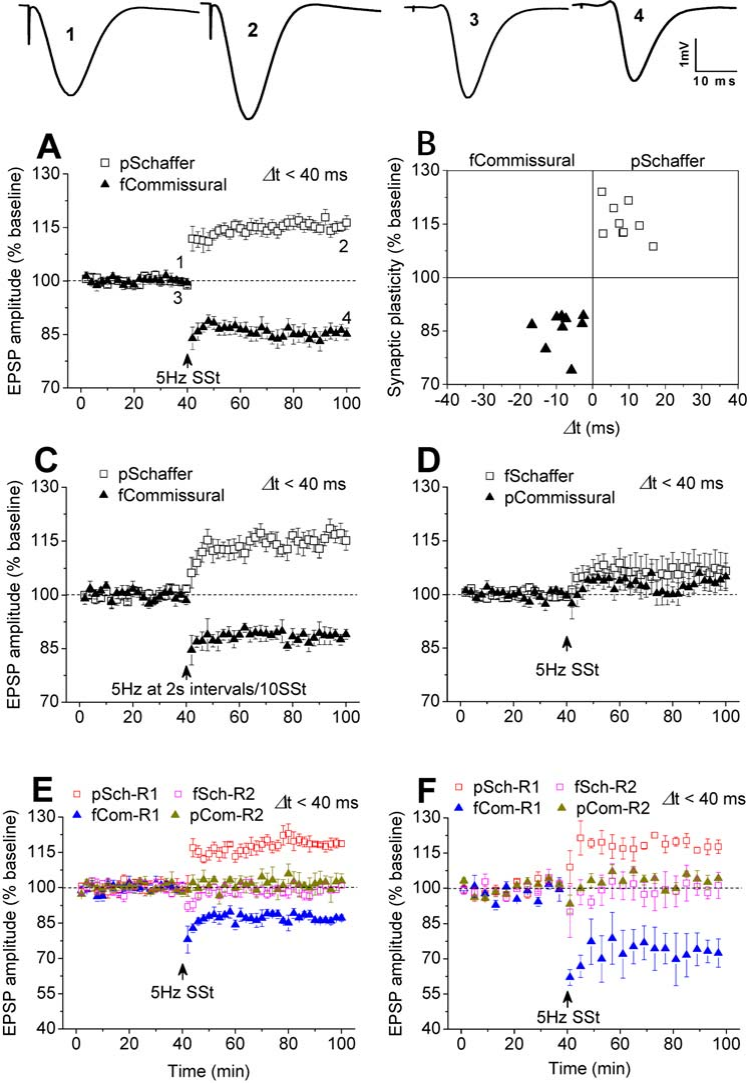
Coincident Schaffer/commissural activity induces LTP and LTD in urethane-anesthetized or freely-moving rats. A, The stimulation to Schaffer preceding to commissural pathway within a timing window of 40 ms induced LTP in the preceding and LTD in the following pathway (n = 9; pSchaffer: 116.6±1.9%; fCommissural: 84.8±2.0%; P<0.001 vs. baseline of pSchaffer or fCommissural). B, Individual data confirmed consistent LTP and LTD in pSchaffer and fCommissural pathways, respectively. C, Similarly, the modified stimulation, consisted of 600 pulses at 5 Hz with 2 s intervals per 10 pulses, to Schaffer preceding to commissural pathway within 40 ms induced Schaffer LTP and commissural LTD, simultaneously(n = 4; pSchaffer: 116.8±1.5%, P = 0.003 vs. baseline; fcommissural: 88.7±2.1%; P = 0.011 vs. baseline). D, However, when the activity sequence was reversed in which Schaffer followed commissural pathway within a window of 40 ms, the stimulation had no significant effect on synaptic efficacy in either pathway (n = 9; fSchaffer: 106.8±4.9%, P = 0.061 vs. baseline; pCommissural: 104.0±3.8%, P = 0.147 vs. baseline). E, In urethane-anesthetized rats, the stimulation to Schaffer preceded commissural pathway induced LTP in the preceding and LTD in the following pathway at R1, but had no effect on synaptic efficacy in the Schaffer followed commissural pathways at R2, within a 40-ms window (n = 3, pSchaffer-R1 (pSch-R1): 118.4±1.5%, P = 0.005 vs. baseline; fCommissural-R1 (fCom-R1): 87.2±1.4%, P = 0.005 vs. baseline; pCommissural-R2 (pCom-R2); 99.8±1.4%, P = 0.437 vs. baseline; fSchaffer-R2 (fSch-R2): 101.8±3.3%, P = 0.352 vs. baseline). F, In freely-moving rats, the stimulation induced LTP in preceding Schaffer and LTD in following commissural pathway (R1); it had no effect on synaptic efficacy in the reversed sequence (R2), within a 40-ms window (n = 4; pSch-R1: 117.5±3.6%, P = 0.002 vs. baseline; fCom-R1: 72.3±7.2%, P = 0.025 vs. baseline; pCom-R2; 99.9±4.8%, P = 0.3987 vs. baseline; fSch-R2: 103.4±3.4%, P = 0.399 vs. baseline).

The above results describe that coincident Schaffer/commissural activity at 5 Hz for 2 min within a 40-ms window induces LTP in both pathways but fails to induce a reliable LTD in pentobarbital-anesthetized rats. As LTP alone may endow flexibility but not stability of the hippocampal network, we tried other strategies. Since Levy and Steward demonstrated the first example of STDP [9], [13] under urethane anesthesia, in which theta activity was enhanced in the hippocampal network [18], [24]–[27] in relative to pentobarbital anesthesia (see Fig. S2), we then tried urethane anesthesia. SSt to Schaffer preceding to commissural pathway (Δt<40 ms) consistently induced LTP in the Schaffer and LTD in the commissural pathway (Fig. 3A, B). Furthermore, we tried a SSt protocol that consisted of 600 pulses at 5 Hz with 2 s intervals per 10 pulses, because this theta-like activity possibly occurs in the hippocampal network under physiological conditions[18]. Similarly, under urethane anesthesia, this ‘natural’ SSt to Schaffer preceding to commissural pathways induced Schaffer LTP and commissural LTD simultaneously in the both pathways (Fig. 3C). This demonstrates that coincident Schaffer and commissural activity can induce LTP and LTD in the hippocampal network *in vivo*, simultaneously.

To our surprise, SSt to Schaffer following to commissural pathway had no significant effect on synaptic efficacy in either pathway (Fig. 3D). This could be due to the properties of the commissural activity in affecting inhibitory function in CA1 as those suggested previously[28]. To confirm this novel finding, we used a tetra-pathway technique to record Schaffer and commissural fEPSP from both the left and right hippocampal CA1s. Under urethane anesthesia, SSt evoked LTP and LTD simultaneously in the Schaffer preceding commissural pathways (Δt<40 ms) on one side of the CA1s (R1, Fig. 3E), and as expected, it had no effect on synaptic efficacy in the Schaffer following commissural pathways (Δt<35 ms) on the other side (R2, Fig. 3E). Furthermore, to produce a state similar as a physiological one, we performed the tetra-pathway experiment with the same protocol in freely-moving rats. Consistent with the above finding, SSt evoked LTP in the preceding Schaffer and LTD in the following commissural pathway on one side of the CA1s (R1, Fig. 3F), but had no effect in the following Schaffer and preceding commissural pathway on the other side (R2, Fig. 3F).

Together, coincident Schaffer/commissural activity can induce either LTP only or LTP/LTD depending on the experimental conditions, the state of which was indicated by the prominent theta activity of the hippocampal network (see Fig. S2). In addition, LTP only or LTP/LTD in the afferent pathways can be rapidly switched to each other by using pharmacological tools such as atropine and carbachol (see Fig. S3), which are known to affect the prominent theta state of the hippocampal network[18].

### This synaptic plasticity was dependent on NMDAR

**Figure 4:**
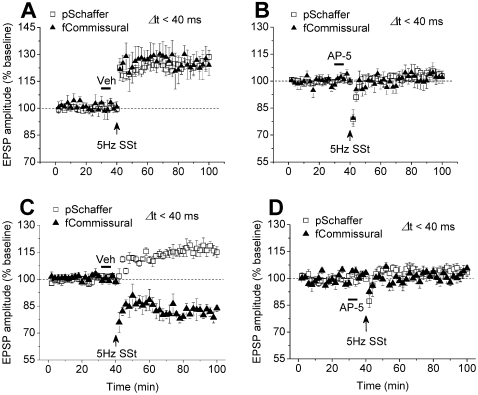
The activity-dependent synaptic plasticity depends on NMDAR. A, Intracerebroventricular infusion of vehicle did not prevent the stimulation to Schaffer preceding commissural pathway (Δt<40 ms) from inducing LTP in both pathways in pentobarbital-anesthetized rats (n = 5; Schaffer: 125.7±3.2%; commissural: 123.9±6.2%; P<0.001 vs. baseline of Schaffer or commissural). B, Infusion of the NMDAR antagonist AP-5 prevented the stimulation to Schaffer preceding to commissural pathway within a 40-ms window from inducing LTP in both pathways (n = 6; Schaffer: 103.6±2.2%, P = 0.349 vs. baseline; commissural: 103.0±4.1%, P = 0.463 vs. baseline). C, Similarly, infusion of vehicle did not affect the stimulation to Schaffer preceding to commissural pathway within a 40-ms window to induce LTP in preceding and LTD in following pathway in urethane-anesthetized rats (n = 6; Schaffer, 116.8±3.2%; commissural, 81.4±2.3%, P<0.001 vs. baseline of pSchaffer or fcommissural). D, Infusion of the NMDAR antagonist AP-5 prevented the stimulation to Schaffer preceding to commissural pathway within a 40-ms window from inducing LTP and LTD (n = 4; Schaffer: 103.8±2.9%, P = 0.163 vs. baseline; commissural: 102.6±2.4%, P = 0.493 vs. baseline).

It is well known that NMDAR is critical for both memory and activity-dependent synaptic plasticity[3]–[5]. Therefore, we further investigated whether this activity-dependent synaptic plasticity shared the same mechanism. By using pentobarbital anaesthesia and protocol in Fig. 2A, infusion of sterile saline (Veh) did not affect LTP in both Schaffer and commissural pathways (Fig. 4A). However, infusion of the NMDAR antagonist AP-5 prevented LTP in both pathways (Fig. 4B). Similarly, by using urethane anaesthesia and protocol in Fig. 3A, infusion of sterile saline (Veh) did not affect Schaffer LTP and commissural LTD (Fig. 4C). However, infusion of the NMDAR antagonist AP-5 prevented Schaffer LTP and commissural LTD (Fig. 4D). Thus, regardless of the anaesthetics, both LTP and LTP/LTD in the hippocampal network were dependent on NMDAR.

### Coincident activity of Schaffer pathways in CA3-CA1 network

**Figure 5:**
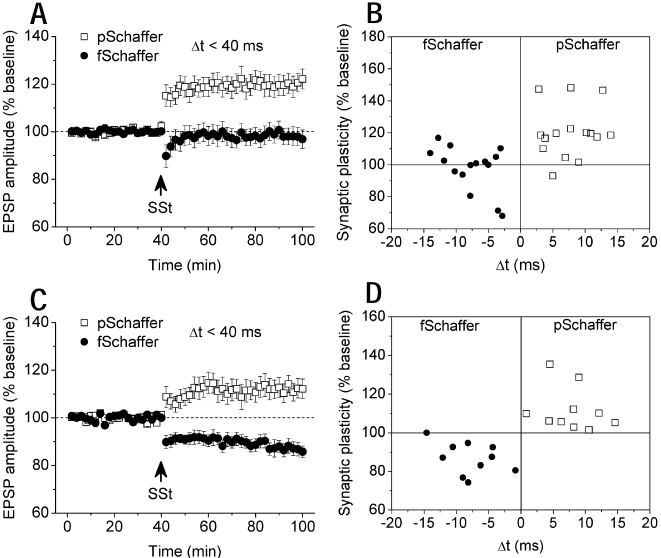
Coincident activity of Schaffer pathways enables synaptic plasticity in CA3-CA1 network. A, In pentobarbital-anesthetized rats, SSt to two Schaffer pathways with one preceding the other (Δt<40 ms) induced LTP in the pSchaffer without affecting synaptic efficacy in the fSchaffer pathway (n = 15; pSchaffer: 120.3±4.0%, P<0.001 vs. baseline; fSchaffer: 97.8±3.5%; P = 0.273 vs. baseline; P<0.001 pSchaffer vs. fSchaffer). p = preceding; f = following. B, Individual data confirmed this finding for activity-dependent LTP. C, In urethane-anesthetized rats, SSt to two Schaffer pathways within a 40-ms window consistently induced LTP in the preceding and LTD in the following pathway (n = 10; pSchaffer: 112.0±3.6%, P = 0.007 vs. baseline; fSchaffer: 86.6±2.6%; P<0.001 vs. baseline). D, Individual data confirmed consistent LTP and LTD in the pSchaffer and fSchaffer pathway, respectively.

The above findings suggest that LTP only or LTP/LTD is determined by the experimental conditions. This could be a feedback to endow the system with flexibility or stability. To confirm this finding, we examined coincident activity of independent Schaffer pathways by one preceding the other (Δt<40 ms) using pentobarbital anaesthesia and techniques described previously[19], [20], [23]. After stable baseline recordings for at least a 40-min period, SSt to Schaffer pathways induced LTP in the preceding pathway, but had no significant effect in the following pathway (Fig. 5, A, B). Similarly, under urethane anaesthesia, SSt to Schaffer pathways with one preceding the other (Δt<40 ms) consistently induced LTP in the preceding and LTD in the following pathway (Fig. 5, C, D). Thus, the experimental condition under pentobarbital and urethane anaesthesia can determine the afferent pathways to produce either LTP only or LTP/LTD simultaneously.

## Discussion

We demonstrated for the first time that coincident activity of afferent pathways induces a dynamic distribution of synaptic plasticity in the hippocampal network, which tends to be sensitive to the state of itself. Since activity-dependent synaptic plasticity is believed to underlie memory[3]–[5], this synaptic plasticity may well contribute to memory process of the network in the aspects of flexibility and stability.

### Coincident pathway/pathway activity for synaptic modification

STDP refers to the phenomenon that coincident pre- and postsynaptic or post- and presynaptic activity within a few tens of milliseconds determines LTP or LTD in the pathway, respectively[11], [12]. It may be a homeostatic mechanism [10], [13] to enable a balance of LTP and LTD in the amygdala *in vitro* [14]. Here, we demonstrated that coincident pathway and pathway activity within a 40-ms window determines LTP only or LTP/LTD in the pathways *in vivo*. For convenience, we termed this type of synaptic plasticity as the timing of afferent pathways dependent plasticity (TADP), because it shares some but not all properties with STDP. First, STDP is either LTP or LTD in one pathway; TADP is LTP only or LTP/LTD simultaneously in afferent pathways of a neural network. Second, timing window of STDP is within a few tens of milliseconds, which is shorter for LTP but wider for LTD in rat hippocampus. Thus it will be difficult to induce LTP and LTD simultaneously; timing window of TADP is within 40 ms, which is the same for LTP and LTD and thus they are induced simultaneously. Third, it is unclear whether STDP is sensitive to experimental conditions *in vivo*; TADP is sensitive to the conditions. Fourth, in STDP, LTP is determined by pre- and postsynaptic activity but LTD is induced by the reversed activity sequence; TADP, on the other hand, means that LTP only or LTP/LTD are induced in both pathways if Schaffer precedes commissural pathway; the reversed activity sequence produces either varied LTP magnitudes or no synaptic plasticity in both pathways. Nevertheless, TADP could very likely be a network form of STDP because both are determined by precise timing and temporal order.

### The sequence rule of coincident pathway/pathway activity

The first example of STDP was found in the dentate gyrus of the hippocampus[9]. Levy and Steward reported that either LTP or LTD in the contralateral pathway to dentate gyrus can be induce by coincident high-frequency stimulation (400 Hz) to ipsilateral preceding or following to contralateral perforant pathway within a 20-ms window[9]. However, it is unknown what occurs in the ipsilateral pathway. Here, we found that either LTP only or LTP/LTD in the pathways to CA1 can be induced by coincident low-frequency stimulation (5 Hz) to ipsilateral Schaffer preceding or following contralateral commissural pathway within a 40-ms window. Commissural LTD is induced in urethane-anesthetized rats if ipsilateral precedes contralateral activity; we found that Schaffer LTP and commissural LTD were induced simultaneously in urethane-anesthetized rats if ipsilateral precedes contralateral activity. Levy and Steward also suggested that the commissural activity is critical for synaptic learning[9], for which we demonstrated that spatial learning was impaired if the commissural activity was disrupted by infusing TTX into bilateral fornix 30 min before, but not after, the spatial learning trainings on each day (see Fig. S4 and Text S1). However, we found that coincident Schaffer following commissural activity had no effect on synaptic efficacy in either urethane-anesthetized or freely-moving rats. This could be meaningful if classic conditioned memory is considered, because the memory can be formed by conditioned stimulation (CS) preceding unconditioned stimulation (US) but not after the reversal of the stimulation sequence (i.e. US-CS). On the other hand, the optimal interval for the memory by CS-US is a few hundreds of milliseconds[11], [12], but the biophysical laws of STDP require the timing of pre- and postsynaptic activity within a few tens of milliseconds. Thus, it is suggested that more circuitry is required to bridge the time gap of behavioural and synaptic learning if STDP is responsible for classic conditioned memories[9]. However, if we assume that CS preceding US within a few hundreds of milliseconds can trigger neural activities for a longer period of time (e.g. 2 min) in a neural network, coincident activity of the CS and US pathways during the overlapping period could allow the time-scale for behavioural events to be scaled down to that required for synaptic learning (e.g. <40-ms for TADP).

### Switching synaptic plasticity distribution in the hippocampal network

Another important finding could be that experimental conditions can enable either LTP only or LTP/LTD simultaneously. The condition in freely-moving rats strongly indicates that LTP and LTD are likely induced simultaneously in one state of the hippocampal network. Furthermore, a stimulation protocol that consisted of 600 pulses at 5 Hz with 2 s intervals per 10 pulses, which possibly occurs in physiological conditions of the hippocampus, induced a similar result in the condition of urethane anaesthesia. In contrast, LTP only was induced in the afferent pathway(s) of the hippocampal network in another state of the hippocampal network in pentobarbital-anesthetized rats. Using pharmacological tools, we demonstrated that the induction of LTP only or LTP/LTD simultaneously in the afferent pathways can be rapidly switched to each other (see Fig. S3).

It is known that outside information more or less crosses the midline to sequentially activate coordinated areas of both brain hemispheres. As a result, sequential activation of the bilateral hippocampal CA3s may lead to the intrinsic timing of Schaffer preceding commissural activity on one side but a reversed activity sequence in the other side and *vice versa*. Also, another intrinsic timing is possible that some Schaffer collaterals precede the others in either side. This could enable LTP and LTD in one side of the CA1s, depending on which side of the CA3s was activated earlier, in conditions of urethane-anaesthesia or wake. In marked contrast, it could allow LTP only in both sides of the CA1s, regardless of which side of the CA3s was activated earlier, in the condition of pentobarbital anaesthesia. These actually are competitive and cooperative phenomena between the afferent pathways of the hippocampal network.

It is known that theta oscillation at 4–8 Hz in the rats is the prominent activity state of the hippocampal network[18]. Evidence shows that the timing of neuronal spiking is constrained to a particular phase of a theta cycle[29]. Thus, the experimental conditions tend to affect theta oscillation in the hippocampal network (see Fig. S2 and Text S1), the state of which in turn switches the afferent pathways to be either LTP only or LTP/LTD simultaneously. According to Wiener's control theory, feedback is critical for stabilizing a dynamic system such as the hippocampal network. Thus, the present TADP findings could be important for memory process of the hippocampal network in the states from time to time. A state of the hippocampal network with LTP only could provide flexibility of the system to learn new information; the other state of the network with LTP and LTD simultaneously could endow stability to the system for retaining acquired information. Although other mechanisms cannot be ruled out, the prominent theta state of the hippocampal network may be a powerful mechanism to rapidly switch flexibility or stability of the network in memory process[18], [29].

However, the network rules of synaptic modification could be even more complex. If coincident activity of two afferent pathways produces TADP, it is still difficult to predict what occurs in the synapses of a neuron by coincident activity among more afferent pathways. Since individual fibres are activating on hundreds or even thousands of the synapses in the dendrite tree of a neuron, it could be a problem to compute which individual synapses will be strengthened or weakened or whether the system is balanced or not. However, the computation could be easier if it follows a simple rule, similar to the TADP finding that strengthens the preceding inputs and weakens the following inputs within a timing window. In contrast, the synapses could be all strengthened or weakened regardless of the sequence only if were they activated coincidently under certain conditions, which would be similar to the cooperative properties required in the HFS or LFS induction of LTP or LTD[3]–[5]. Furthermore, since TADP was also dependent on the activation of NMDAR, similar to the mechanism for the HFS or LFS induction of LTP or LTD[3]–[5], a stability hypothesis could be that forms of activity-dependent synaptic plasticity such as those with the properties of frequency, timing and sequence may work in a concert to endow stability to the hippocampal network for retaining acquired information while flexibility for learning new information, in which the system could be rapidly switched to each other.

## Materials and Methods

### Animals

Experiments were carried out on male Sprague-Dawley rats (inbred strain, Animal House Center, Kunming Medical College, Kunming), weighing 200–300 g. Animals were group-housed with free access to water and food with a 12 h light/dark cycle and a thermoregulated environment. Animal care and experimental protocols were approved by the Chinese Academy of Sciences, PR China.

### Electrophysiology

Animals were under pentobarbital (60 mg/kg, i.p.) or urethane (1.5 g/kg, i.p.) anaesthesia, and core temperature was maintained at 37±0.5°C. Recordings of fEPSPs were made from the CA1 stratum radiatum in response to ipsilateral Schaffer and contralateral commissural stimulation by using techniques described previously[19]–[23]. Electrodes were made by gluing together a pair of twisted Teflon-coated 90% platinum/10% iridium wires (50 µm inner diameter, 75 µm outer diameter, World Precision Instruments, USA). Test Schaffer and commissural fEPSPs were evoked alternately at an interval of 30 s and at a stimulus intensity adjusted to give 50% fEPSPs amplitude of maximum. To induce LTP or LTD, sequential stimulation (SSt) that consisted of 600 pulses at 5 Hz, or at 1 and 10 Hz was delivered to ipsilateral Schaffer and contralateral commissural pathways with stimulation timing between both inputs indicated by the inter-peak intervals (Δt) of the fEPSPs (see *inset*, Fig. 2). A modified SSt was also tested, which was consisted of 600 pulses at 5 Hz with 2 s intervals per 10 pulses. LTP and LTD during the last 5 min of recordings was measured as mean±s.e.m.% of the baseline fEPSPs amplitude recorded over at least a 40-min period. Each point in figures was the average of 4 sweeps (2 min) in anaesthetized or the average of 8 sweeps (4 min) in freely-moving rats.

In all cases, we found independent dual or tetra pathways and then used them for experiments, in which paired-pulse stimulation (40 ms inter-pulse intervals) produced less than 10% paired-pulse potentiation or depression in the followed pathway[19]–[21], [23]. The stimulation and recording electrodes were located with the stereotaxic parameters (stimulation electrode: AP = −4.8 mm, MR/L = ±3.8±0.5 mm and DV = −3.0 mm; Recording electrode: AP = −3.8 mm, MR/L = ±2.8 mm and DV = −2.5 mm) relative to bregma and skull. In some animals, the tetra electrodes were fixed by dental cement and experiments were performed in a home cage in freely-moving rats.

### Drug and treatment

Drugs used in these experiments were sodium channel blocker tetrodotoxin (TTX) and the NMDA receptor antagonist AP-5, purchased from Sigma. The drugs were dissolved in sterile saline (Veh).

Under pentobarbital anesthesia (60 mg/kg, i.p.), rats were implanted stainless steel guide cannulas (26 gauge, 11 mm) that were affixed to the skull with dental cement by using techniques similar to those described[30]. The cannulas were located in the lateral cerebral ventricle (AP = 0.5 mm, ML = 1.5 mm and DV = −4.0 mm) or in the bilateral fornix (AP = −1.1, MR/L = ±0.7 and DV = −3.0 mm) relative to bregma and skull. Intracerebroventricular (i.c.v.) or intra-fornix injections were made over a 6-min period by a syringe pump, connected to injectors (32 gauges) by polyethylene tubing. AP-5 (10 mM, 6 µl for 6 min, i.c.v.) was infused 10 min before the conditioning stimuli. TTX (5 ng, 0.5 µl per cannula for 6 min, projecting 1 mm beyond the cannulas by polyethylene tubing) was infused after the baseline recordings to identify the independence of the Schaffer and commissural fEPSPs. The cannula placement was verified in each animal by histological examination of the brain after methylene blue injection (0.5 µl)[30], and only the data obtained from rats with correctly inserted cannula were included in the statistical analysis.

### Data analysis

The number of rats used was indicated by *n*. LTP or LTD comparisons were made by using *t*-test compared with 40-min baseline. The magnitude of LTP or LTD was the average of the last 10 min recordings. Between-groups comparisons were conducted by one-way ANOVA followed by least significance difference (LSD) test (SPSS 13.0). Significance level was set at *p*<0.05.

## Supporting Information

Text S1 (0.06 MB DOC)

Figure S1 The timing and frequency dependence of coincident Schaffer and commissural activity A, SSt to the Schaffer (Sch) or the commissural pathway (Com) alone failed to evoke synaptic plasticity in either pathway (n = 3, Schaffer: 99.6±2.5%, P = 0.270 vs. baseline; commissural: 100.1±2.5%, P = 0.487 vs. baseline). B, SSt (600 pulses) at 1 Hz to the Schaffer preceding to the commissural pathway within a 40-ms window had no effect on synaptic efficacy in either pathway (n = 3, pSchaffer: 103.3±3.0%, P = 0.381 vs. baseline; fCommissural: 95.5±2.7%, P = 0.119 vs. baseline). p = preceding; f = following. C, SSt (600 pulses) at 10 Hz to the Schaffer preceding to the commissural pathway within a 40-ms window failed to affect synaptic efficacy in either pathway (n = 3, pSchaffer: 97.1±2.4%, P = 0.282 vs. baseline; fCommissural: 100.9±3.8%, P = 0.459 vs. baseline).
(0.03 MB TIF)

Figure S2 The hippocampal activity states under experimental conditions EEG traces were recorded from the hippocampal CA1 (upper-left panel) in pentobarbital- (n = 7) and urethane-anaesthetized (n = 7) or freely-moving rats (n = 4). Calibration bars: Vertical 0.1 mV; Horizontal 200 ms. The hippocampal EEG power was higher in urethane-anaesthetized and much higher in awake rats (A), which was correlated to the magnitude of LTD in following commissural pathway but not to that of LTP in preceding Schaffer pathway (B) (F(2,72) = 83.326, *P<0.05, ** P<0.01 pentobarbital vs. urethane or awake, two-way ANOVA followed by LSD).
(0.04 MB TIF)

Figure S3 Pharmacological tools rapidly switch the distribution of synaptic plasticity A and B, Under pentobarbital anesthesia, we demonstrated that coincident Schaffer preceding commissural activity enabled LTP only in the both pathways. However, after carbachol treatment for 20 min, the theta activity of the hippocampal network was enhanced (n = 4 each group, baseline vs. carbachol, F(1,31) = 11.145, **P = 0.003; frequency vs. frequency, F(3,31) = 69.606, P<0.001; two-way ANOVA), and SSt to Schaffer preceding to commissural activity within a 40-ms window induced Schaffer LTP and commissural LTD simultaneously (n = 4, pSchaffer: 116.8±2.0%, P = 0.002 vs. baseline; fCommissural: 89.4±1.5%, P = 0.004 vs. baseline, one-tailed t-test). C and D, Under urethane anesthesia, we demonstrated that coincident Schaffer preceding commissural activity enabled Schaffer LTP and commissural LTD, simultaneously. However, after atropine treatment for 20 min, the theta activity of the hippocampal network was reduced (n = 4 each group, baseline vs. atropine, F(1,31) = 4.284, *P = 0.049; frequency vs. frequency, F(3,31) = 163.085, P<0.001; two-way ANOVA), and SSt to Schaffer preceding commissural pathway within a 40-ms window induced reliable LTP in both pathways (n = 4, pSchaffer: 111.7±1.1%, P = 0.001 vs. baseline; fCommissural: 124.2±5.1%, P = 0.037 vs. baseline).
(0.04 MB TIF)

Figure S4 Commissural activity is required for spatial learning. A, Bilateral inactivation of the fornix with tetrodotoxin (TTX) 30 min before daily learning trainings significantly impaired spatial learning on days 1–5 (n = 10 vehicle, n = 7 TTX; day vs. day, F(5,101) = 20.104, P<0.001; vehicle vs. TTX, F(1,101) = 48.428, P<0.001, two-way ANOVA; F(1,15) = 2.301, P = 0.150 vehicle vs. TTX on day 6, one-way ANOVA). B, TTX was infused 30 min after the learning task on each day, but rats still learned the spatial learning tasks very well as indicated by short latencies in escape (n = 9 vehicle; n = 9 TTX; day vs. day, F(5,96) = 34.089, P = 0.001; vehicle vs. TTX, F(1,96) = 0.008, P = 0.927; day×group F(5,96) = 0.057, P = 0.998; two-way ANOVA). C and D, TTX had no effect on swim speed during the spatial learning task compared with vehicle control.
(1.90 MB TIF)
