# Supplementary material for: Coincident Activity of Converging Pathways Enables Simultaneous Long-Term Potentiation and Long-Term Depression in Hippocampal CA1 Network In Vivo
Source: PLoS One. 2008 Aug 6;3(8):e2848. doi: 10.1371/journal.pone.0002848 (PMC2475662; doi:10.1371/journal.pone.0002848)
Supplement: Text S1 — (0.06 MB DOC) [file pone.0002848.s001.doc]

**Supporting information**

**Methods and references**

**Electroencephalogram (EEG) recording**. Using techniques described previously[1], the EEG was monitored from the hippocampal recording electrode. The spectral power of the EEG was measured after fast Fourier transformation of sweeps of 5 s duration by using the Scope software of the PowerLab 4e (USA).

**Drug and treatment**. The drugs used in experiments were the sodium channel blocker tetrodotoxin (TTX, 5 ng per cannula, intra-fornix infusion), the mAChR agonist carbachol (0.2 mg/kg, i.p.) and antagonist atropine (5 mg/kg, i.p.) purchased from Sigma. The drugs were dissolved in sterile saline (Veh).

**Guide cannulas implantation.** Under pentobarbital anesthesia (60 mg/kg, i.p.), rats were implanted stainless steel guide cannulas (26 gauge, 11 mm) that were affixed to the skull with dental cement by using techniques similar as those described[2]. The cannulas were located in the bilateral fornix (AP = - 1.1, MR/L = ± 0.7 and DV = - 3.0 mm) relative to bregma and skull. In behavioral study, the animals were allowed at least 7 days of recovery from surgery at home cage.

Intra-fornix infusions were made over a 6-min period by a syringe pump, connected to injectors (32 gauges) by polyethylene tubing. In behavioral studies, TTX (5 ng, 0.5 μl per cannula for 6 min, projecting 1 mm beyond the guide cannulas by polyethylene tubing) was infused 30 min before or after daily spatial learning trainings. The cannula placement was verified in each animal by histological examination of the brain after methylene blue injection (0.5 μl)[2], and only the data obtained from rats with correctly inserted cannulas were included in statistical analysis.

**Morris water maze**. The formation of spatial memory was examined by using procedures and techniques similar as those described[3,4]. The apparatus consisted of a circular pool (60 cm height, 250 cm diameter) filled with water at 25 ± 1°C to a depth of 20 cm, and the water surface was covered with floating black resin beads. Yellow curtains were drawn around the pool and contained distinctive visual marks served as distal cues. The maze was considered to consist of four quadrants, and a submerged Perspex platform (14 × 14 cm) was placed in the middle of one quadrant for all training trials. Rats always faced the pool wall when placed into the maze at four starting positions (NE, NW, SE, and SW). Twenty-four hours before the spatial learning task, animals were allowed to adapt to the maze for a 120-s free swim. Animals were then trained in the spatial learning task by 4 trials per day with 10 min inter-trial intervals for 6 consecutive days. Spatial learning was measured by escape latencies of the rats onto the hidden platform by using an automatic tracking system[4].

**Data analysis.** The number of rats used was indicated by *n*. LTP or LTD comparisons were made by using *t*-test compared with 40-min baseline. The magnitude of LTP or LTD was the average of the last 10 min recordings. Between-groups comparisons were conducted by one-way ANOVA followed by least significance difference (LSD) test (SPSS 13.0). EEG comparisons among groups were conducted by one-way ANOVA followed by LSD test. In behavioral test, data comparisons were carried out by one-way or two-way ANOVA. Significance level was set at *p* < 0.05.

**References**

1. Xu L, Holscher C, Anwyl R, Rowan MJ (1998) Glucocorticoid receptor and protein/RNA synthesis-dependent mechanisms underlie the control of synaptic plasticity by stress. Proc Natl Acad Sci U S A 95: 3204-3208.

2. Dong Z, Han H, Wang M, Xu L, Hao W, et al. (2006) Morphine conditioned place preference depends on glucocorticoid receptors in both hippocampus and nucleus accumbens. Hippocampus 16: 809-813.

3. Morris RG, Anderson E, Lynch GS, Baudry M (1986) Selective impairment of learning and blockade of long-term potentiation by an N-methyl-D-aspartate receptor antagonist, AP5. Nature 319: 774-776.

4. Yang Y, Cao J, Xiong W, Zhang J, Zhou Q, et al. (2003) Both stress experience and age determine the impairment or enhancement effect of stress on spatial memory retrieval. J Endocrinol 178: 45-54.
