# Supplementary figures and images for: Coincident Activity of Converging Pathways Enables Simultaneous Long-Term Potentiation and Long-Term Depression in Hippocampal CA1 Network In Vivo
Source: PLoS One. 2008 Aug 6;3(8):e2848. doi: 10.1371/journal.pone.0002848 (PMC2475662; doi:10.1371/journal.pone.0002848)

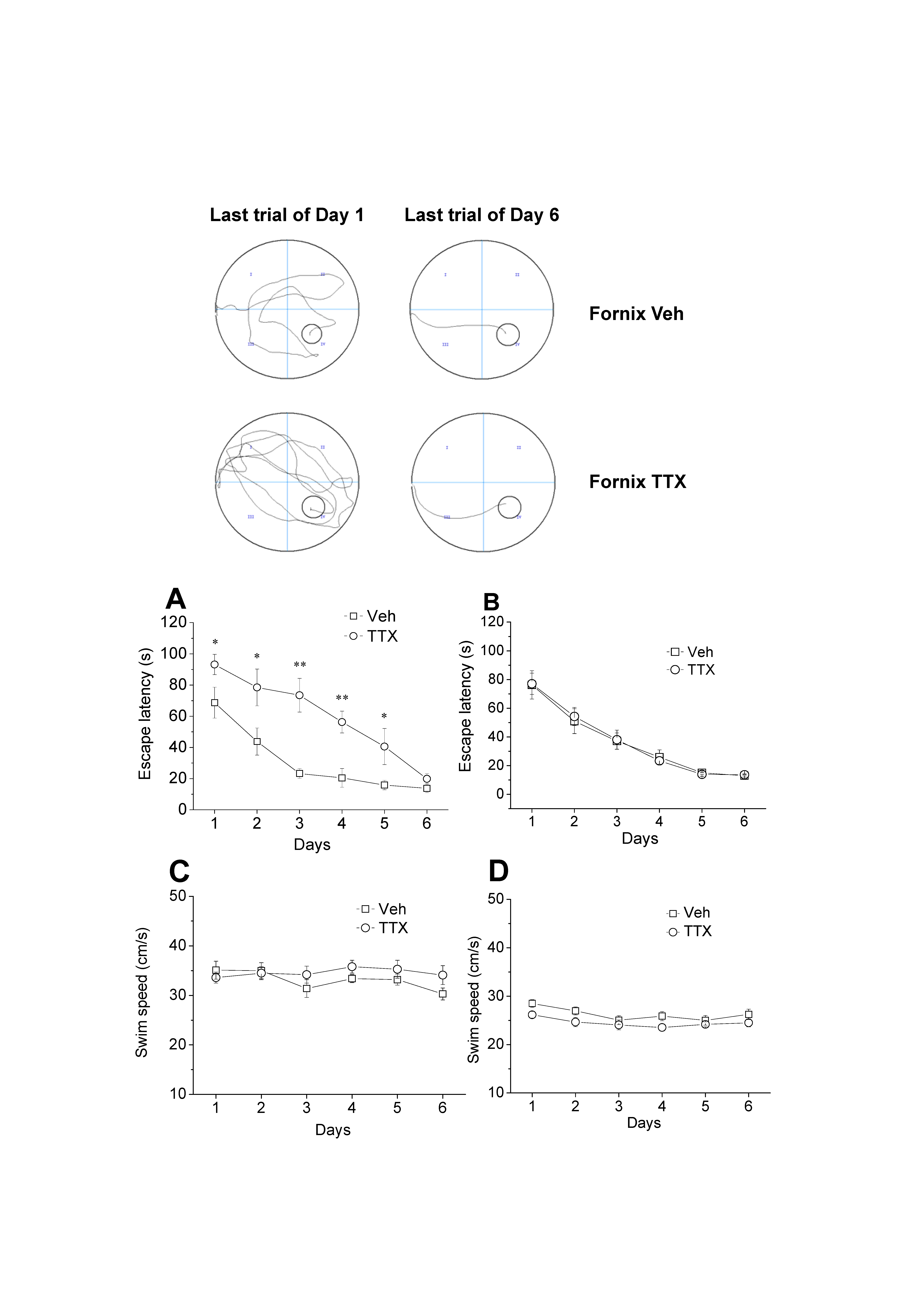

Supplement: Figure S4 — Commissural activity is required for spatial learning. A, Bilateral inactivation of the fornix with tetrodotoxin (TTX) 30 min before daily learning trainings significantly impaired spatial learning on days 1–5 (n = 10 vehicle, n = 7 TTX; day vs. day, F(5,101) = 20.104, P<0.001; vehicle vs. TTX, F(1,101) = 48.428, P<0.001, two-way ANOVA; F(1,15) = 2.301, P = 0.150 vehicle vs. TTX on day 6, one-way ANOVA). B, TTX was infused 30 min after the learning task on each day, but rats still learned the spatial learning tasks very well as indicated by short latencies in escape (n = 9 vehicle; n = 9 TTX; day vs. day, F(5,96) = 34.089, P = 0.001; vehicle vs. TTX, F(1,96) = 0.008, P = 0.927; day×group F(5,96) = 0.057, P = 0.998; two-way ANOVA). C and D, TTX had no effect on swim speed during the spatial learning task compared with vehicle control. (1.90 MB TIF) [file pone.0002848.s005.tif]
